# Responses to combined abiotic and biotic stress in tomato are governed by stress intensity and resistance mechanism

**DOI:** 10.1093/jxb/erw285

**Published:** 2016-07-19

**Authors:** Christos Kissoudis, Sri Sunarti, Clemens van de Wiel, Richard G.F. Visser, C. Gerard van der Linden, Yuling Bai

**Affiliations:** Wageningen UR Plant Breeding, Wageningen University & Research Centre, PO Box 386, 6700AJ, Wageningen, The Netherlands

**Keywords:** Callose, cell death, ethylene, invertase, R-gene resistance, stress severity.

## Abstract

Stress conditions in agricultural ecosystems can occur at variable intensities. Different resistance mechanisms against abiotic stress and pathogens are deployed by plants. Thus, it is important to examine plant responses to stress combinations under different scenarios. Here, we evaluated the effect of different levels of salt stress ranging from mild to severe (50, 100, and 150mM NaCl) on powdery mildew resistance and overall performance of tomato introgression lines with contrasting levels of partial resistance, as well as near-isogenic lines (NILs) carrying the resistance gene *Ol-1* (associated with a slow hypersensitivity response; HR), *ol-2* (an *mlo* mutant associated with papilla formation), and *Ol-4* (an *R* gene associated with a fast HR). Powdery mildew resistance was affected by salt stress in a genotype- and stress intensity-dependent manner. In susceptible and partial resistant lines, increased susceptibility was observed under mild salt stress (50mM) which was accompanied by accelerated cell death-like senescence. In contrast, severe salt stress (150mM) reduced disease symptoms. Na^+^ and Cl^−^ accumulation in the leaves was linearly related to the decreased pathogen symptoms under severe stress. In contrast, complete resistance mediated by *ol-2* and *Ol-4* was unaffected under all treatment combinations, and was associated with a decreased growth penalty. Increased susceptibility and senescence under combined stress in NIL-Ol-1 was associated with the induction of ethylene and jasmonic acid pathway genes and the cell wall invertase gene LIN6. These results highlight the significance of stress severity and resistance type on the plant’s performance under the combination of abiotic and biotic stress.

## Highlight

Salt stress severity differentially affected partial powdery mildew resistance. Stress combination uniquely resulted in accelerated senescence. *mlo*-based and R-gene-mediated resistance maintained robustness across salt treatments.

## Introduction

Plants in their natural environment are continuously exposed to a variety of stress factors, both abiotic and biotic, and thus have evolved a multitude of defence mechanisms in order to maintain their fitness (Roux *et al.*, 2014; Mickelbart *et al.*, 2015). Under natural conditions, both the timing and the intensity of the stressors can vary; thus, appropriate fine-tuning of the defence responses is required to minimize detrimental effects on plant fitness (Des Marais and Juenger, 2010; Brown and Rant, 2013). Stress interactions between abiotic and biotic agents are projected to become more prevalent with the observed and predicted changes in global climate patterns. The average temperature increase and decrease in precipitation especially in regions with temperate climates (Dai, 2013; Cook *et al.*, 2015) can accelerate agricultural land deterioration leading to yield losses (Lobell *et al.*, 2011). In the same way, increased temperatures can result in geographic expansion of pathogens and enhanced fecundity, increasing the chances for host range expansion and the rise of more virulent strains (Harvell *et al.*, 2002; Garrett *et al.*, 2006).

Field crops are grown under the same variable conditions; however, as they are bred and selected under relatively controlled conditions, several trade-offs might have been overlooked that can result in negative interactions under field conditions (Brown and Rant, 2013; Hückelhoven *et al.*, 2013; McGrann *et al.*, 2014). It is thus of great importance to examine plant responses to combinations of abiotic and biotic stress factors, important variables that are relevant to crop yields (Soliman and Kostandi, 1998; Kissoudis *et al.*, 2014).

Studies aimed at elucidating interactions between abiotic and biotic stress responses are limited. The majority of these studies conclude that there is a negative impact of abiotic stress (mostly drought and salinity stress) on pathogen resistance (Suzuki *et al.*, 2014); however, positive effects have also been reported on resistance to foliar pathogens in a plant- and/or pathogen-specific manner (Kissoudis *et al.*, 2014). Plant response and performance under different stress levels is not linear (Maggio *et al.*, 2007; Malkinson and Tielbörger, 2010; Cheng *et al.*, 2013; Muralidharan *et al.*, 2014) and this can significantly impact the phenotypic responses under stress combinations. An early study on maize susceptibility to smut disease (*Ustilago maydis*) under different salt stress concentrations concluded that disease severity decreased when salt stress increased to 9 dS m^–1^, and an inverse relationship between disease susceptibility and plant Cl^−^ content was observed (Soliman and Kostandi, 1998). Resistance to pathogens can also be differentially affected by the imposition of various types of abiotic stress. For example, rice resistance to *Magnaporthe grisea* mediated by dominant resistance (R)-genes was not affected by cold stress or abscisic acid (ABA) application (Koga *et al.*, 2004). In contrast, heat stress was shown to impact negatively the resistance controlled by the Arabidopsis R-genes, *SNC1* and *RPS4*, in an ABA-dependent manner (Mang *et al.*, 2012). In barley, *mlo*-based recessive resistance to powdery mildew was compromised during recovery after drought stress (Baker *et al.*, 1998).

Functional molecular studies have added pieces to the puzzle of interactions between abiotic and biotic stress signalling components with the identification of several genes and transcription factors involved in stress crosstalk (Liu *et al.*, 2012; Yokotani *et al.*, 2013). ABA appears to be a central modulator of the regulatory crosstalk, directly impacting biosynthesis of salicylic acid, the major regulatory hormone for defence responses against biotrophic pathogens (Yasuda *et al.*, 2008; De Torres Zabala *et al.*, 2009). In some cases, successful pathogenesis of a number of pathogens involves the manipulation of the ABA pathway (De Torres-Zabala *et al.*, 2007; Kazan and Lyons, 2014). On the other hand, enhanced callose deposition, a significant line of defence enhancing plant penetration resistance against pathogens, is positively regulated by the ABA pathway (Cao *et al.*, 2011). Thus ABA–defence signalling interactions appear to be complex, and the outcome is greatly affected by the host and pathosystem as well as by the timing of infection (Ton *et al.*, 2009; Chen *et al.*, 2013).

Our research is focused on the response and performance of tomato under combined salinity stress and powdery mildew infection caused by *Oidium neolycopersici*. We have previously reported a negative impact of salinity stress (100mM NaCl) on powdery mildew resistance in a tomato introgression line (IL) population exhibiting partial resistance to powdery mildew (Kissoudis *et al.*, 2015). In this study, we advance a step further, examining the effects of different salt stress levels representative of mild and severe stress on powdery mildew resistance. We selected the above-mentioned ILs with contrasting resistance. In addition, we used near-isogenic lines (NILs) which carry monogenic resistance genes, namely *Ol-1* (no gene characterized yet), *ol-2* (an *mlo* mutant), and *Ol-4* [an NBS (nucleotide-binding site) R-gene]. The resistance conferred by *Ol-1*, *ol-2*, and *Ol-4* is associated with a slow hypersensitive response (HR), papilla formation, and a fast HR, respectively (Bai *et al.*, 2005). Our results indicated a significant interaction of powdery mildew resistance with salt stress severity that was dependent on the resistance mechanism. The detailed coverage of the different variables in terms of both stress intensity and type of disease resistance gene provides significant insights on realistic scenarios of abiotic–biotic stress interactions, and potentiates efficient and targeted crop breeding for combined stress tolerance.

## Materials and methods

### Plant material

ILs 2-3, 3-2, 4-2, 4-3, 6-2, 6-3, 8-2, 9-1, and 10-4 harbouring introgressions of *Solanum habrochaites* LYC4 in the genetic background of *Solanum lycopersicum* cv. Moneymaker (MM) were selected on the basis of their salt tolerance and/or powdery mildew resistance (Kissoudis *et al.*, 2015). Additionally, the NILs NIL-Ol-1, NIL-ol-2, and NIL-Ol-4 were used.

The pathogenic fungus *O. neolycopersici* originated from infected commercial tomato plants (Lindhout *et al.*, 1993) and was maintained on MM plants in a greenhouse compartment at 20±3 °C with 70±15% relative humidity.

### Experimental conditions and treatments

Experiments were carried out at the Unifarm greenhouse facilities of Wageningen University & Research Centre. The photoperiod regime was 16h light and 8h dark. Greenhouse air humidity was 70%. Additional lighting (100W m^−2^) was used if the incoming shortwave radiation was <200W m^−2^.

Two independent experiments were carried out in two different years in spring (April–May). Plants were grown in pots filled with vermiculite and were irrigated with half-strength Hoagland’s nutrient solution with or without NaCl till leaching of the solution at regular intervals, avoiding accumulation of nutrients and NaCl. To ensure that, the electrical conductivity (EC) of the leachate after watering was periodically evaluated.

In the first experiment, plants of all the above-mentioned genotypes were evaluated for their susceptibility to powdery mildew under different salt stress regimes. Three-week-old plants (*n*=4) were watered with a solution containing different concentrations of NaCl (0, no salt stress; 50, 100, and 150mM NaCl). Eight days after the initiation of salt treatments, plants were inoculated with powdery mildew by uniformly spraying a suspension of 5×10^4^ conidia ml^−1^. Plants were grown for another 25 days post-inoculation (dpi) in order to observe secondary infection symptoms.

In the second experiment, only NIL-Ol-1, NIL-ol-2, NIL-Ol-4, and the recurrent parent cv. MM were evaluated. Three-week-old plants (*n*=4) were watered with 0, 50, and 150mM NaCl. In this case, 8 d after the salt treatments half of the plants were spatially isolated and were not sprayed with powdery mildew, resulting in three treatments: no salt stress/not inoculated, salt stress/not inoculated, and salt stress/inoculated. Plants were grown for another 20 d after inoculation.

### Plant performance evaluation under salt stress and powdery mildew infection

Chlorophyll content was measured using a SPAD-502 meter (Minolta, Osaka, Japan) at the third and fourth leaf counting from the bottom, on the fifth day after pathogen inoculation, before symptom appearance. Fresh and dry weights were measured as described previously (Kissoudis *et al.*, 2015). The disease severity was expressed as the disease index (DI) on a scale from 0 to 5, according to Kissoudis *et al.* (2015), assessed at 10, 15, and 25 dpi for the first experiment and at 15 dpi for the second experiment. In addition to DI, a measure of the visual stress response was introduced to describe the accelerated senescence phenotypes observed at the later stages of infection under salt stress: 0=healthy plant; 1=0.1–10% of foliar area affected; 2=10–20% of area affected with yellowing and moderate wilting; 3=20–30% of area affected with severe wilting; 4=30–50% of area affected with severe wilting and moderate leaf abscission; and 5= >50% of area affected with severe wilting and leaf abscission.

### Ion content

Sampling for ion content determination differed between the two experiments. In the first experiment the fourth leaf counting from the bottom was sampled at 10 dpi, shortly after symptom appearance, in order to assess the relationship between disease severity and ion concentration. In the second experiment, the top five leaves were sampled at 20 dpi, the end-point of the experiment, in order to examine differences in actively growing tissues, potentially linked to growth performance, and to avoid the dying bottom leaves of susceptible genotypes under combined stress conditions. The ion analysis included Na^+^, Cl^−^, K^+^, PO_4_^3−^, SO_4_^2−^, Mg^2+^, and Ca^2+^, and quantification was performed as described previously (Kissoudis *et al.*, 2015).

### Histological analyses of *in situ* callose deposition

Leaf disks (1.3cm in diameter) were sampled from leaflets of the fourth leaf counting from the bottom on the third day after pathogen inoculation, from the middle of the leaflets on both sides of the central vein. Staining was carried out in 24-well plates, with leaf disks placed with their abaxial side up. Callose deposition visualization was performed according to Ton *et al.* (2005) and Luna *et al.* (2011) with slight modifications. Leaf disks were placed in 96% ethanol to remove chlorophyll and, after a 1min wash in 0.07M K_2_HPO_4_ (pH 9), stained for 2h in 0.05% (w/v) aniline blue in 0.07M K_2_HPO_4_ (pH 9) at room temperature. Leaf disks were subsequently mounted on glass slides with 70% glycerol. Callose was quantified from digital photographs as the number of white pixels (fluorescence, callose intensity) relative to the total number of plant-derived pixels.

### Gene expression and pathogen quantification with qPCR

Leaflets for the gene expression analyses were sampled 6 dpi from the third and fourth leaf counting from the bottom, before pathogen mycelium growth was visible. Leaflets for pathogen quantification were sampled 14 dpi inoculation from the fourth and fifth leaf counting from the bottom, when pathogen growth from the primary infection was highest.

RNA for gene expression analyses was isolated with the RNeasy plant mini kit (Qiagen). Plant and fungal DNA for pathogen quantification analyses was extracted with the DNeasy plant mini kit (Qiagen). RNA was treated with DNase I (Invitrogen) to eliminate residual DNA. cDNA synthesis was performed with 1 μg of RNA template using an iScript™ cDNA Synthesis Kit (Bio-Rad). Quantitative real-time PCR was conducted using the iQ SYBR Green supermix (Bio-Rad) and the CFX96 Real-Time system (Bio-Rad).

The reaction mix contained 5 µl of 2× iQ SYBR GREEN super mix, 1 µl of forward primer (3 µM), 1 µl of reverse primer (3 µM), and 3 µl of cDNA (or DNA, 20ng) template, into a final volume of 10 µl. Thermocycling conditions were 95 °C for 3min, followed by 40 cycles of 95 °C for 30s and 60 °C for 30s. Primers used for fungal quantification were Fw-On-CGCCAAAGACCTAACCAAAA and Rv-On-AGCCAAGAGATCCGTTGTTG (Gao *et al.*, 2014). Primers for tomato elongation factor 1α (EF) were Fw-EF-GGAACTTGAGAAGGAGCCTAAG and Rv-EF-CAACACCAACAGCAACAGTCT. The primers used for the expression analysis of selected tomato genes are provided in Supplementary Table S1 at *JXB* online. Relative expression was calculated using the 2-ΔΔCt method (Livak and Schmittgen, 2001).

### Statistical analysis

Experiments were carried out in a split plot design with treatments (salt stress, powdery mildew, and combined stresses) as main plots and the genotypes as subplots (*n*=4). Statistical analyses were performed using Genstat 15th edition. Multiple comparisons between means were performed using protected LSD (*P*≤0.05). Correlations between traits were calculated using the Pearson correlation coefficient (*P*≤0.1).The relationship between elemental concentration (independent variable) and disease severity (dependent variable) was examined with multivariate regression analysis.

## Results

### Effect of salt stress severity on powdery mildew resistance

To examine the effect of salinity stress intensity on powdery mildew resistance, we evaluated the response of nine *S. habrochaites* LYC4 ILs, selected from a previous study (Kissoudis *et al.*, 2015), in which they were shown to carry introgressions for salinity tolerance and/or powdery mildew resistance. We applied three levels of stress, which are considered to be low (50mM), intermediate (100mM), and high (150mM NaCl) salinity stress for most crops including tomato (Munns and Tester, 2008).

**Fig. 1.**
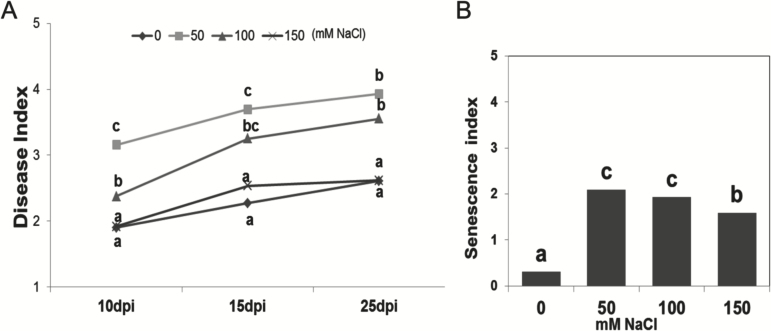
(A) Disease index averaged across the LYC4 ILs and the recurrent parent MM under powdery mildew without salt (0mM NaCl) and in combination with 50, 100, and 150mM NaCl, measured at 10, 15, and 25 days post-inoculation (dpi). (B) Senescence index across the same genotypes and treatments at 15 dpi. Statistically significant differences (*P*≤0.05) between salinity levels (within each time point of measurement) are designated with different letters.

Powdery mildew disease severity was on average the highest at 50mM NaCl, but decreased at higher salt concentrations (Fig. 1A). In particular, at 10 dpi the average disease index was 65% higher in plants grown at 50mM NaCl compared with no salt stress conditions. This effect of salt stress on the DI was more pronounced in ILs that exhibited a higher level of resistance, such as ILs 3-2, 4-3, and 9-1 (Supplementary Fig. S1).

A unique response was observed under combined salt stress and powdery mildew infection, with leaves initially exhibiting increased epinasty, even before visible pathogen growth. Shortly after the mildew appearance (9–10 dpi), yellowing and wilting were observed, which in more susceptible genotypes led to up to 50% leaf abscission. These accelerated senescence and leaf abscission phenotypes were expressed as a visual stress index (scale 0–5). Similar to the DI, the senescence index was highest at 50mM NaCl (Fig. 1B).

**Fig. 2.**
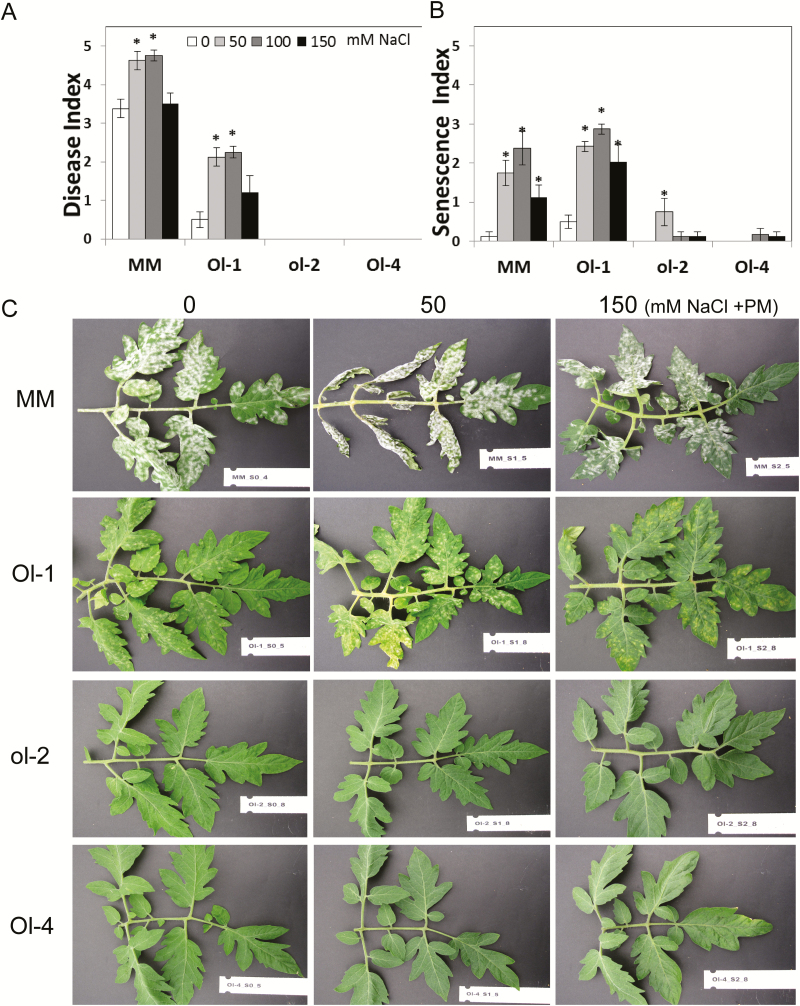
(A) Disease and (B) senescence index of NIL-O-1, NIL-ol-2, and NIL-Ol-4 (written as Ol-1, ol-2, Ol-4 in the figure) and the recurrent parent MM under powdery mildew without salt (0mM NaCl) and in combination with 50, 100, and 150mM NaCl, measured at 15 days post-inoculation. (C) Leaf phenotypes under different powdery mildew and combined stress treatments. Asterisks denote statistically significant pairwise differences (*P*≤0.05) between powdery mildew without NaCl and each of the combined stress [powdery mildew+NaCl) treatments for each genotype (*n*=4; error bars represent the SEM)].

In addition to the LYC4 ILs, NIL-Ol-1, NIL-ol-2, and NIL-Ol-4, conferring monogenic resistance to powdery mildew through different mechanisms (Bai *et al.*, 2005), were evaluated under the same treatment scheme. The responses under combined stress were largely disparate among the different genotypes. Resistance in NIL-Ol-1 was partially compromised at 50mM and 100mM NaCl stress, while resistance was partially restored at 150mM NaCl stress. Additionally, NIL-Ol-1 exhibited accelerated senescence and runaway cell death, leading to leaf abscission. Resistance in NIL-ol-2 and NIL-Ol-4, in contrast, was not affected by salt stress at any salt stress level. No wilting or senescence symptoms were observed in either of the genotypes (Fig. 2A–C; Supplementary Fig. S2).

### Relationship of different disease resistance responses to ion content and gene expression

**Fig. 3.**
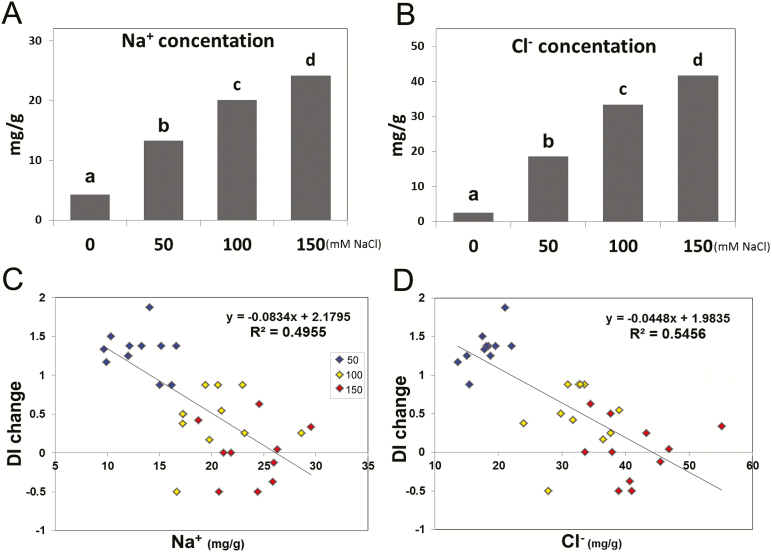
(A, B) Averaged concentrations of Na^+^ and Cl^−^ across the LYC4 ILs and the recurrent parent MM under powdery mildew without salt and in combination with salt stress. Statistically significant differences (*P*≤0.05) are designated with different letters. (C, D) Regression analysis between Na^+^ and Cl^−^ concentration and disease index (DI) change across the different salinity treatments in combination with powdery mildew. The dependent variable DI was calculated by subtracting DI under salt stress conditions from that under non-salt stress conditions (only powdery mildew infection). *R*^2^=percentage of variance of DI change explained by the Na^+^ and Cl^−^ concentration.

The ions Na^+^, Cl^−^, K^+^, PO_4_^3−^, SO_4_^2−^, Mg^2+^, and Ca^2+^ were measured at 10 dpi to determine any possible relationship between leaf ion concentration and disease severity under salt stress. Both shoot Na^+^ and Cl^−^ concentrations increased linearly with increased NaCl application (Fig. 3A, B). K^+^ and SO_4_^2−^ concentrations were decreased under salt stress, with no differences observed between the different salinity levels, while no significant changes were observed for PO_4_^3−^, Mg^2+^, and Ca^2+^ (Supplementary Fig. S3).

We examined whether there was a causal relationship between the decreased DI and increased ion contents at higher stress levels using multiple stepwise regression. We used LYC4 ILs and NIL-Ol-1 along with the recurrent parent MM, in which resistance was affected by salt stress (Figs 1, 2). DI under salt stress conditions subtracted from that under non-salt stress conditions (only powdery mildew infection–DI change) was used as the dependent variable, which was strongly correlated with Na^+^ and with Cl^−^ concentrations. The Na^+^ and Cl^−^ concentration increase accounted for 50% and 55% of the variation in DI change, respectively (Fig. 3C, D). The addition of the rest of the ions to the model led to a slight decrease in the variance explained (46.7%), and separately (without Na^+^ and Cl^−^) these ions did not contribute significantly to variation in DI either (*P*=0.068, 18% variance explained).

Correlations for the different growth, ion content, and disease susceptibility traits measured were calculated within each stress level (Supplementary Table S2). Under powdery mildew infection (no salt stress), DI was weakly negatively correlated with FW and DW (*r*= –0.4 and –0.39, respectively, *P*<0.1) and SO_4_^2−^ concentration (*r*= –0.52). At 50mM NaCl, these correlations with DI were no longer significant, which was then negatively correlated with Ca^2+^ concentration (*r*= –0.47). The senescence index was positively correlated with FW and DW (*r*= 0.5 and 0.52, respectively) and negatively correlated with PO_4_^3−^ concentration (*r*= –0.5). No significant correlations were observed between DI and any of the traits measured at 100mM NaCl. Finally at 150mM NaCl, the negative effect of Na^+^ and Cl^−^ accumulation on plant performance was apparent, with a negative correlation observed for shoot Na^+^ and (especially) Cl^−^ concentrations with FW and DW (Supplementary Table S2).

Examining expression markers for major hormonal and other biochemical pathways involved in resistance to stress and defence to pathogens in selected ILs (the powdery mildew-resistant IL3-2 and 9-1 and the salt-tolerant IL8-2, Kissoudis *et al.*, 2015) revealed a noticeable trend in the underlying molecular responses under different intensities of salt stress and powdery mildew infection. Averaged across genotypes, the expression of ACCase, encoding a biosynthetic enzyme of ethylene, was highest under the combination of mild salt stress (50mM) NaCl. A similar (but less strong) response was observed for the jasmonic acid (JA) biosynthesis and response genes AOS and LOXD, respectively. PR1a expression was significantly up-regulated under combined stress. On the other hand, induction of NCED, an ABA biosynthesis enzyme, was modest under both mild and severe salt stress combinations with powdery mildew (Supplementary Fig. S4).

### Performance of NILs under salt stress, powdery mildew, and their combination

The above-mentioned results of LYC4 ILs indicated a significant effect of the genotype and the stress intensity on powdery mildew resistance under salt stress. In a second experiment, we focused on the NILs carrying the Ol-genes and the response to 50mM and 150mM NaCl, representing the most contrasting responses. Plants were exposed to non-stress, a single stress (salt or powdery mildew), as well as to combined stress.

**Fig. 4.**
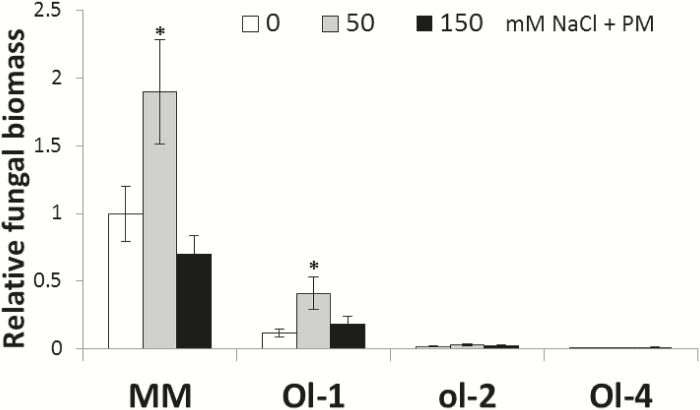
Relative *Oidium neolycopersici* fungal biomass in MM and NIL-O-1, NIL-ol-2, and NIL-Ol-4 (written as Ol-1, ol-2, Ol-4 in the figure) under powdery mildew infection alone and in combination with 50mM and 150mM NaCl. Values are normalized with that of MM under powdery mildew infection (no salt stress). Asterisks denote statistically significant pairwise differences (*P*≤0.05) between powdery mildew (0mM NaCl+PM) and each of the combined stress treatments (50mM NaCl+PM and 150mM NaCl+PM) for an individual genotype (*n*=4; error bars represent the SEM).

Similarly to the first experiment, under mild salt stress (50mM NaCl) increased susceptibility and senescence were observed in MM and NIL-Ol-1, while under severe salt stress (150mM NaCl) this effect was reversed. The resistance of NIL-ol-2 and NIL-Ol-4 was not affected in any of the treatments, and senescence was hardly increased. Fungal biomass was quantified and the results confirmed the visual DI scores (Fig. 4).

**Fig. 5.**
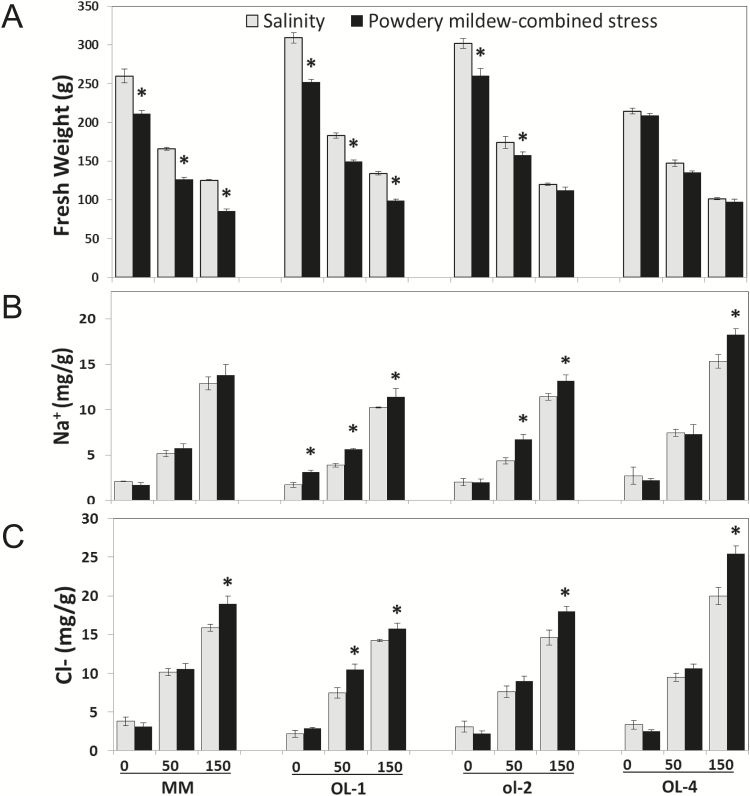
(A) Above-ground biomass (FW) of NIL-O-1, NIL-ol-2, and NIL-Ol-4 (written as Ol-1, ol-2, Ol-4 in the figure) and the recurrent parent MM under salt stress (0, 50, and 150mM NaCl) or powdery mildew alone, and their combination. Level 0 for salinity stress corresponds to stress-free control conditions, while level 0 for powdery mildew-combined stress corresponds to powdery mildew infection alone (no salt stress). (B) Na^+^ and (C) Cl^−^ concentration of Ol-lines and MM under the same treatment scheme Asterisks denote statistically significant differences (*P*≤0.05) between salinity and powdery mildew-combined stress, within each salt level for an individual genotype (*n*=4; error bars represent the SEM).

A single stress factor (either increased salinity or powdery mildew infection) resulted in a significant penalty on plant performance. MM and all NILs showed decreased plant FW under salt treatment (Fig. 5A). Upon powdery mildew infection only (no salt stress), a reduction of 20% in terms of FW was observed for MM and NIL-Ol-1, and 15% for NIL-ol-2 compared with control non-infected plants (no salt stress). NIL-Ol-4 on the other hand showed no significant reduction in FW. Under combined stress (both salt stress and powdery mildew infection), MM and NIL-Ol-1 exhibited a further significant decrease in biomass of 15% and 12% compared with salt stress only. NIL-ol-2 and NIL-Ol-4 were less affected—a 5% and 4% decrease in biomass in 50mM NaCl with powdery mildew (significant only for NIL-ol-2)—while they maintained their biomass to similar levels with salt stress alone in 150mM NaCl with powdery mildew (Fig. 5A).

Under the assumption that ion concentration was related to growth performance of the plants, sampling for ion analysis was carried out at the end of the experiment. The top five leaves were collected to avoid sampling of senescing (or abscised) leaves from MM and NIL-Ol-1 plants. Both Na^+^ and Cl^−^ concentrations were slightly increased on most occasions under combined salt stress with powdery mildew compared with salt stress alone (Fig. 5B, C). Small differences were observed between genotypes, with the increase of Na^+^ and Cl^−^ concentration under combined stress being higher in NIL-Ol-1 at 50mM NaCl and in NIL-ol-2 and NIL-Ol-4 at 150mM NaCl compared with MM. No significant differences were observed for the other ions, except for a higher concentration of SO_4_^2−^ and, to a lesser extent, K^+^ and Ca^2+^ in NIL-Ol-1, and K^+^ in NIL-ol-2 under powdery mildew and combined stress compared with non-stress and salt stress only (Supplementary Fig. S5).

### Callose deposition

**Fig. 6.**
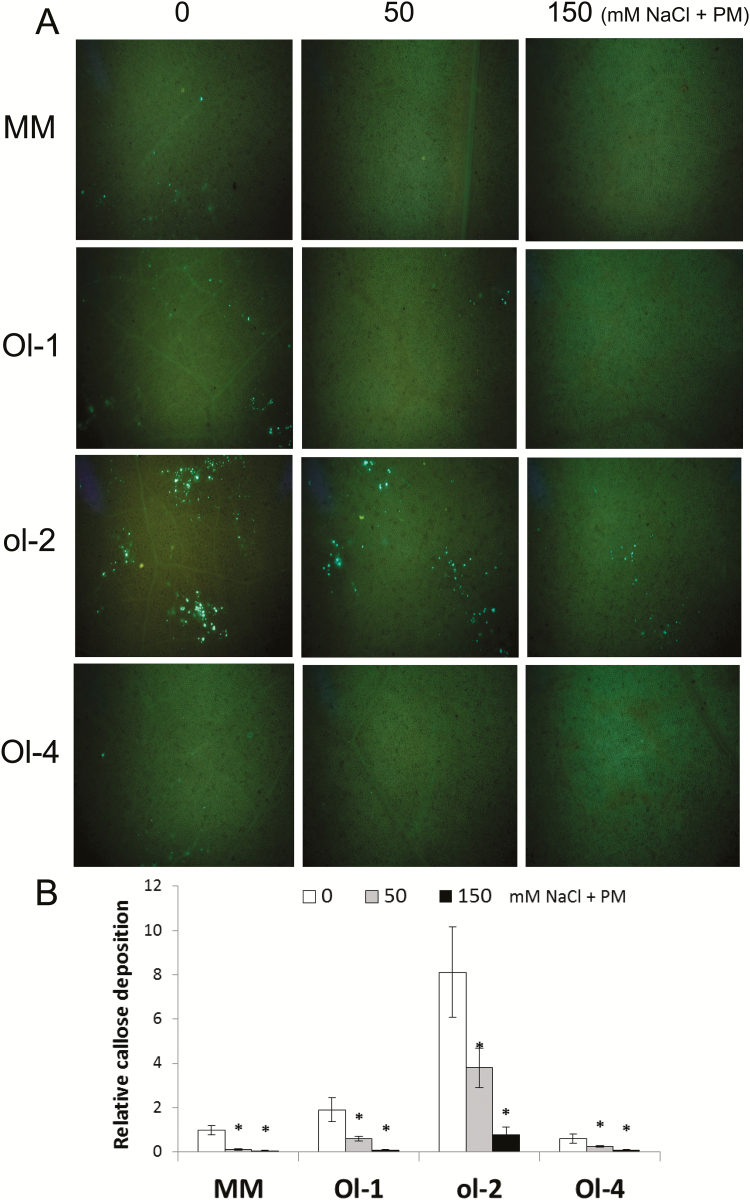
(A) Callose deposits in leaves as visualized with UV microscopy after aniline blue staining. (B) Quantification of callose deposition relative to MM under powdery mildew infection (no salt stress). Asterisks denote statistically significant pairwise differences (*P*≤0.05) between powdery mildew (0mM NaCl+PM) and each of the combined stress treatments (50mM NaCl+PM and 150mM NaCl+PM) for an individual genotype.

*In situ* callose deposition at the leaf level was evaluated by aniline blue staining and examined by UV epifluoresence microscopy. Callose deposition is an important penetration resistance mechanism against pathogens, and powdery mildew in particular, and is associated with ol-2-based resistance. Under powdery mildew infection, NIL-ol-2 exhibited the highest intensity of callose deposits. Far fewer callose deposits were observed in NIL-Ol-1 and MM, and these were almost absent in NIL-Ol-4. Under salt stress with powdery mildew, decreased callose depositition was observed in all genotypes. Callose deposition under 50mM and 150mM was almost abolished in MM and NIL-Ol-1 and was much lower in NIL-ol-2, especially under 150mM NaCl (Fig. 6A, B).

### Gene expression analyses

**Fig. 7.**
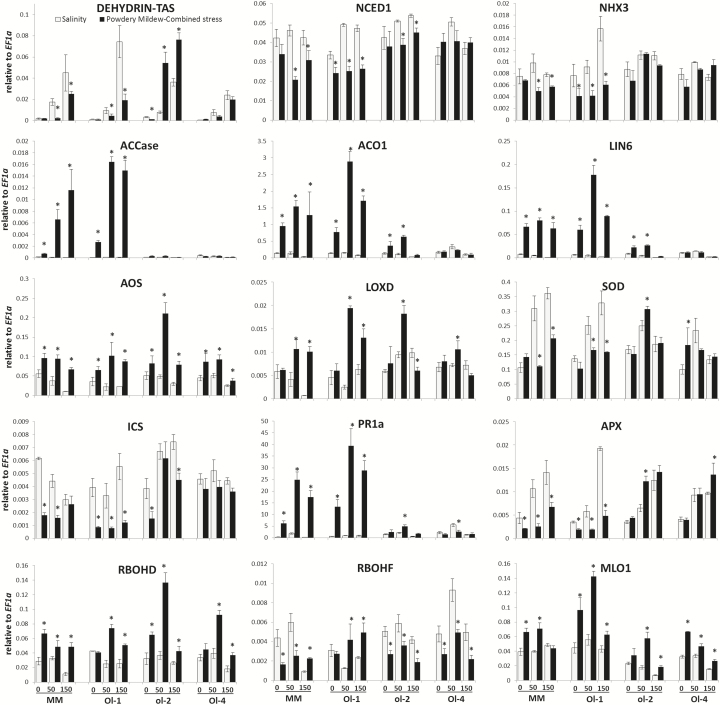
Expression of marker genes for hormonal, abiotic, and biotic stress signalling pathways in MM, and NIL-Ol-1, NIL-ol-2, and NIL-Ol-4 (written as Ol-1, ol-2, Ol-4 in the figure), relative to *EF1a*, which was used as a housekeeping gene. The treatment and labelling scheme are the same as in Fig. 5. Asterisks denote statistically significant differences (*P*≤0.05) between salinity and powdery mildew-combined stress, within each salt level and for an individual genotype (*n*=4; error bars represent the SEM).

In order to link the responses of MM and NILs to specific pathways, we measured expression of marker genes in defence, hormone, reactive oxygen species (ROS), antioxidant, and ion homeostasis pathways (Fig. 7; Supplementary Fig. S6). For the ABA pathway, no significant expression changes were observed for the ABA-synthesizing enzyme NCED under salt stress compared with control conditions, with the exception of NIL-Ol-1 showing an up-regulation. However, a reduction of NCED expression (2-fold for MM and NIL-Ol-1) was observed under combined stress versus salt stress alone. A significant reduction in the expression of *DHN-TAS* was observed under combined stress for MM and NIL-Ol-1 (ranging from 2- to 7-fold), while an induction was observed in NIL-ol-2.

Regarding the ethylene pathway, a dramatic induction of expression of ethylene biosynthesis genes *ACCase* and *ACCox* was observed in MM and NIL-Ol-1 under combined stress (ranging from 50- to 400-fold for *ACCase*), as well as of the ethylene-responsive *Chitinase9*. Similarly, the JA biosynthesis and signalling genes *AOS* and *LOXD* were highly up-regulated in MM and NIL-Ol-1. The pathogenesis-related gene *PR1a* was induced up to 70- and 45-fold in MM and NIL-Ol-1, respectively, while *NPR1* was similarly induced in both, albeit to a lesser extent. On the other hand, *ICS* expression was significantly reduced under powdery mildew and in combination with salt stress in MM, NIL-Ol-1, and NIL-ol-2. The cell wall invertase (CWI) LIN6 was induced up to 230-fold for MM and 50-fold for NIL-Ol-1.

Contrasting responses were observed for *RBOHD* and *RBOHF*, genes involved in ROS signalling . On the one hand, *RBOHD* expression was increased under powdery mildew and combined stress. The induction was higher in NIL-ol-2 and NIL-Ol-4 (2-fold higher compared with MM and NIL-Ol-1). On the other hand, *RBOHF* expression was reduced under powdery mildew and combined stress in all genotypes, except for NIL-Ol-1, in which it was induced. Differential responses were also observed among the different genotypes for the antioxidant enzymes APX and SOD. Expression of both genes was significantly reduced under powdery mildew and combined stress in MM and NIL-Ol-1, while in NIL-ol-2 and NIL-Ol-4 their expression remained stable or was slightly increased.

Expression of the *MLO* gene (a negative regulator of disease resistance) was increased (especially in NIL-Ol-1) under powdery mildew and combined stress, while expression of the Na^+^–H^+^ antiporter NHX3 was decreased under combined stress in comparison with salt stress only, with the strongest reduction (2.5-fold) observed in NIL-Ol-1. The rest of the genes examined did not show significant changes in expression or showed no conclusive expression patterns in relation to the different treatments or genotypes (Supplementary Fig. S6).

## Discussion

The results presented here address two dimensions related to the complexity of plant responses under combinatorial stress: the abiotic stress intensity and the resistance mechanism. Both variables are of great importance for crop cultivation practices, as plants are exposed to variable stress intensities during their lifetime and the cultivars deployed often have different mechanisms of resistance.

### Mild salt stress has the most significant impact on susceptibility and partial resistance to powdery mildew

In our study, the susceptible control MM and the LYC4 ILs with partial resistance to powdery mildew showed comparable responses to single and combined stresses. Under mild (50mM) and moderate (100mM) salt stress the observations are in agreement with many studies reporting a negative effect of abiotic stress on disease resistance (Yasuda *et al.*, 2008; Prasch and Sonnewald, 2013; Kissoudis *et al.*, 2014). Interestingly, mild salt stress most severely promoted disease susceptibility and leaf wilting and senescence. Severe salt stress (150mM), on the other hand, partly reversed this effect, with susceptibility for some genotypes being even lower than for plants under no salt stress. This still came at the expense of overall plant performance and growth, as severe salt stress imposed a severe growth penalty. These observations are of great importance for agricultural practices and the potential threat of abiotic and biotic stress combinations for plant productivity. Mild stress conditions are the most prevalent in agricultural lands, and therefore are highly relevant (Vadez *et al.*, 2013). The reduction of susceptibility under high salt stress has limited relevance for agricultural production because of the severe growth penalty; most of the major crops are considered glycophytes and have reduced growth and productivity by at least 50% at salt levels of 150mM NaCl (equalling EC values of 15–20) (Munns and Tester, 2008).

Apart from its effect on powdery mildew susceptibility, the stress combination of salt and powdery mildew resulted in another unique response, namely accelerated leaf wilting, senescence, and leaf abscission, which was not observed under salt stress or powdery mildew alone. Cell death and apoptosis are shared in the responses to the single stress factors, and finely regulated (Torres *et al.*, 2005; Miller *et al.*, 2009; Demidchik *et al.*, 2014; Petrov *et al.*, 2015). The stress combination may have disrupted these balances, resulting in an uncontrolled cell death/senescence phenotype. Such a response is an important aspect of the negative interaction of defence pathways when plants are exposed to these stresses at the same time, and such a response can be detrimental for plant productivity (Gregersen *et al.*, 2013).

### A direct fungal toxicity role for Na^+^ and Cl^−^?

A unique component differentiating salt stress from other abiotic stresses such as drought or heat is Na^+^ and Cl^−^ accumulation. This often has a toxic effect on the plant, but is toxic to the fungus as well. NaCl is known to be an antifungal agent (Blomberg and Adler, 1993) and it could potentially exert a direct toxic effect on fungal growth after accumulation in the plants. Our results point to a direct influence of Na^+^ and Cl^−^ on pathogenicity as observed between the different salt stress levels. This is in line with the many examples of reduction of fungal pathogenicity by metal accumulation (Poschenrieder *et al.*, 2006; Fones *et al.*, 2010), and a similar trend is observed for smut disease and NaCl accumulation in maize (Soliman and Kostandi, 1998). The decreased susceptibility observed under severe stress conditions may therefore be a unique aspect of salt stress that cannot be extrapolated to other abiotic stresses such as drought. Yet increased drought severity also appeared to decrease susceptibility to powdery mildew in garlic mustard (Enright and Cipollini, 2011) and to *Sclerotinia sclerotiorum* (a necrotrophic fungus) and *Pseudomonas syringae* pv. *tabaci* (a hemi-biotrophic bacterial pathogen) in *Nicotiana benthamiana* (Ramegowda *et al.*, 2013).

In addition to Na^+^ and Cl^−^, a weak negative correlation was evident between SO_4_^2−^ and Ca^2+^ concentration and increased disease resistance. Though no strong conclusions can be drawn, these observations highlight the importance of the nutritional status of the plant in the incremental build-up (or breakdown) of basal quantitative resistance. Both SO_4_^2−^ and Ca^2+^ nutrition improve disease resistance (Kruse *et al.*, 2007; Jiang *et al.*, 2013), and thus perturbation of their homeostasis under combined stress potentially contributes to derailing plant defences.

### Robustness and decreased fitness cost of *mlo* and R-gene-based resistance to powdery mildew under salt stress combination

In contrast to the relatively uniform response of LYC4 ILs under combined stress, stark differences were observed between the NILs conferring monogenic resistance through different mechanisms. While NIL-ol-2 and NIL-Ol-4 had a robust resistance phenotype under all treatments of combined stress with maintenance of (almost) complete resistance and no accelerated senescence response, resistance in NIL-Ol-1 succumbed under combination with salt stress, resembling the response of the LYC4 ILs.

The phenotypic differences were reflected in the gene expression patters. Similar gene expression patterns were shown in the susceptible MM, LYC4 ILs with partial resistance, and NIL-Ol-1 with complete resistance to powdery mildew. The massive induction of ethylene biosynthesis genes in NIL-Ol-1 which was absent in NIL-ol-2 and NIL-Ol-4, may underlie its increased susceptibility and senescence under stress combination in a biphasic way, enhancing susceptibility of living cells and eventually leading to cell necrosis and leaf abscission. Ethylene signalling has been demonstrated to be a prerequisite for symptom development after pathogen infection in tomato (O’Donnell *et al.*, 2003). The very strong induction observed uniquely under combined stress in this study is likely to accelerate senescence and leaf abscission, potentiating the action of H_2_O_2_ and resulting in programmed cell death (PCD) processes (Sakamoto *et al.*, 2008; Bar-Dror *et al.*, 2011), in line with our observations of accelerated senescence in NIL-Ol-1 under combined stress.

The very strong induction of the CWI LIN6 specifically under powdery mildew and combined stress in NIL-Ol-1 may additionally contribute to the observed phenotypes. CWIs are induced after pathogen infection (Moghaddam and Van Den Ende, 2012); however, their contribution to plant defence during pathogenesis is still not known. Several studies report a positive contribution of CWIs in plant resistance (Essmann *et al.*, 2008; Bonfig *et al.*, 2010; Sonnewald *et al.*, 2012); however, in tomato an opposite observation is reported, with CWIs contributing to symptom development in response to *Xanthomonas campestris* pv. *vesicatoria* (Kocal *et al.*, 2008). Co-silencing of Lin6 and Lin8 CWIs in tomato reduces the induction of pathogenesis related (PR-) genes together with pathogenesis symptom development (Kocal *et al.*, 2008). In addition to the up-regulation in response to pathogens, PR proteins have been involved in processes such as senescence and leaf abscission (Van Loon *et al.*, 2006). The very high CWI induction under stress combination in NIL-Ol-1 along with PR1a (not observed in individual stress treatments) would therefore seem more likely to be a result of and response to (higher) pathogen infection and contribute to symptom development and the acceleration of senescence and leaf abscission.

Performance in terms of biomass was (also) significantly impacted by powdery mildew and combined stress, in line with the notion that induction of defence responses against pathogens comes at a cost (Bolton, 2009). However combined stress exhibited even greater cost than powdery mildew and salt stress alone, which was most pronounced in MM and NIL-Ol-1. On the other hand, NIL-Ol-4 did not show any additional fitness cost under stress combination. The fitness cost in MM and NIL-Ol-1 might be due to increased senescence and a potential down-regulation of photosynthesis in response to the activation of defence hormone signalling, especially of ethylene and JA (Bilgin *et al.*, 2010). Down-regulation of adaptive and protective mechanisms involved in abiotic stress tolerance such as ABA signalling (evidenced by the reduction in DHN-TAS expression) and the reduced expression of APX and SOD under combined stress potentially contributed to decreased tolerance (Faize *et al.*, 2011; Muñoz-Mayor *et al.*, 2012), while the latter might also have decreased the threshold for the cell death responses observed as increased senescence (Yao and Greenberg, 2006; Stael *et al.*, 2015). Na^+^ and Cl^−^ concentrations in the leaves were slightly more increased under stress combination than under only salt stress, which may additionally contribute to the augmented growth penalty under these conditions. NIL-Ol-4, however, did not show any fitness cost despite exhibiting the highest increase in Na^+^ and Cl^−^ under combined stress compared with salt stress alone.

### What are the causal mechanisms underlying the contrasting responses of NILs with different resistance mechanisms?

The question remains of whether the alterations observed in hormone and energy signalling (ethylene/JA signalling and CWI induction) are the cause or the consequence of the dramatic differences observed in the differential response of NIL-Ol-1, NIL-ol-2, and NIL-Ol-4. Resistance of the three NILs is based on completely different mechanisms. The *Ol-1* gene, probably a non-NBS-LRR (leucine-rich repeat) gene, confers incomplete dominant resistance characterized by a multiple-cell delayed cell death (slow HR; Seifi *et al.*, 2011). The cell death in NIL-Ol-1 can retard but not completely stop fungal development (Bai *et al.*, 2005; Li *et al.*, 2007). The *ol-2* gene (an *mlo* mutant) confers non-race-specific resistance through formation of papillae at the fungal penetration sites. The *Ol-4* gene is an *Mi-1*-like gene (an NBS-LRR gene) and confers complete race-specific resistance associated with single-cell death (fast HR; Bai *et al.*, 2005; Li *et al.*, 2007; Seifi *et al.*, 2011). Thus, the inhibition of fungal penetration (*ol-2*) and growth immediately after penetration (*Ol-4*) did not allow the pathogen to interfere with intracellular signalling and metabolic processes, explaining the lack of induction of the aforementioned pathways.

Early signalling events in both abiotic and biotic stress include Ca^+^ fluxes and ROS generation whose specific signatures orchestrate downstream responses (Segonzac *et al.*, 2011; Demidchik *et al.*, 2014) and pre-invasive defence responses such as callose deposition. The expression of two antioxidant enzyme genes was reduced and RBOH gene expression altered in the NILs under stress combination, indicating that these signatures may be changed and the deployment of defence mechanisms may be different. These changes might have altered the ROS footprint in NIL-Ol-1 and have led to defence breakdown and accelerated cell death. Ol-1-mediated resistance is prone to breakdown when cellular homeostatic mechanisms are perturbed, as shown in ALS-silenced plants, while resistance conferred by *Ol-4* was not affected by the same manipulation (Gao *et al.*, 2014).

Callose deposition was also significantly affected under combined stress. It was almost completely diminished in NIL-Ol-1 under combined stress. Although callose deposition is not the major contributor to Ol-1-mediated resistance against powdery mildew (Li *et al.*, 2007, 2012), the decreased callose deposits might have additionally contributed to accelerated pathogen growth under combined stresses. Callose deposits were much higher in NIL-ol-2 and became very low at higher salt stress levels (150mM NaCl). Callose deposition regulation is complex and, while it has been shown to be positively regulated by ABA signalling (Ton *et al.* 2009), under salt stress conditions multiple factors might be affected such as altered redox status and vesicular trafficking, both important regulatory and functional components for callose formation (Hamaji *et al.*, 2009; Luna *et al.*, 2011).

R-gene [of the toll interleukin 1 receptor (TIR)-NBS-LRR class] function was shown to be affected by abiotic stress, heat in particular (Mang *et al.*, 2012), and to be regulated by proteins involved in heat stress tolerance (Hubert *et al.*, 2009). Ol-4-mediated resistance was not affected at all by salt stress in our experiments, which may be due to the different plant response to salt stress compared with heat. In addition, *Ol-4* is a coiled-coil (CC)-NBS-LRR gene, which confers resistance through different routes compared with TIR-NBS-LRR genes (Teh and Hofius, 2014), such as being autophagy independent. R-gene-mediated effector-triggered immunity (ETI) is characterized by compensatory relationships between hormone signalling pathways (Tsuda *et al.*, 2009), and its defence output is stronger and more prolonged compared with PAMP-triggered immunity (PTI) (Tsuda *et al.*, 2013), thus more robust and less prone to negative regulation from environmental or genetic factors (Cui *et al.*, 2015).

### Conclusions and breeding routes for achieving robust combined powdery mildew and salt stress tolerance in tomato

We conclude that the impact of combined salinity and powdery mildew on tomato plants is dependent both on the salt stress severity and the mechanism of disease resistance. Negative interactions were generally observed under mild salt stress, relevant for most agricultural scenarios, including increased powdery mildew susceptibility, leaf senescence, and decreased biomass. These effects were partly reversed under severe salt stress, but this significantly impacted plant biomass. HR-based disease resistance appears to be the most robust in our experiments in terms of both resistance and overall plant performance under combined stress. Since R-gene resistance appears to be more stable to environmental and genetic perturbations, the additional pyramiding of salt tolerance genes to R-gene-mediated resistance is expected to be more straightforward as fewer interactions can be expected (Kissoudis *et al.*, 2014). A drawback is that pathogens can easily overcome R-gene resistance, thus pyramiding multiple R-genes is essential as well. The recessive *ol-2* gene has the advantage that it is race non-specific, thus more stable over time. However, *mlo*-based resistance may be accompanied by increased senescence at the later stages of plant development (Piffanelli *et al.*, 2002), and this can be further accelerated by abiotic stress. Fine-tuning of ethylene biosynthesis/response might be a key in mitigating the adverse effects of abiotic and biotic stress combination in genotypes with partial disease resistance. Down-regulation of ethylene biosynthesis significantly increased grain yield of maize under drought (Habben *et al.*, 2014) and can potentially contribute to increased crop resilience under scenarios of biotic stress combinations.

The results reported here may be transferable and translated to other crops, as the core stress tolerance/defence response genetic regulation appears to be universal, despite the existence of species-specific responses. However, each stress (abiotic or biotic) has some unique properties (e.g. toxic effects of Na^+^ and Cl^−^ on pathogens are unique to salt stress) that need to be taken into account. Moreover, studies should be extended to cover the entire life cycle of plants, as plant age might significantly influence the phenotypic response, and senescence in particular.

## Supplementary data

Supplementary data are available at *JXB* online

Figure S1. Disease index of the LYC4 ILs, the recurrent parent MM, and the donor parent *S. habrochaites* LYC4 under powdery mildew alone and in combination with 50, 100, and 150mM NaCl measured at 15 dpi.

Figure S2. Whole-plant phenotypes of NILs and MM under salt stress (0, 50, and 150mM NaCl) alone and in combination with powdery mildew.

Figure S3. Averaged K^+^, SO_4_^2−^, Mg^2+^, and Ca^2+^ concentration of the LYC4 ILs and MM under powdery mildew alone and in combination with 50, 100, and 150mM NaCl.

Figure S4. Gene expression heatmap of selected LYC4 ILs and the recurrent parent MM under powdery mildew stress without salt and in combination with salt stress (50mM and 150mM NaCl).

Figure S5. K^+^, SO_4_^2−^, Mg^2+^, and Ca^2+^ concentration of Ol-lines and MM.

Figure S6. Expression analysis of additional genes related to hormone and stress signalling in MM, NIL-Ol-1, NIL-ol-2, and NIL-Ol-4.

Table S1. Primers used for expression analyses with qRT-PCR.

Table S2. Genetic correlations of traits measured in the LYC4 ILs and the recurrent parent MM under powdery mildew individually and in combination with different levels of salt.

Supplementary Data
