# Supplementary material for: Responses to combined abiotic and biotic stress in tomato are governed by stress intensity and resistance mechanism
Source: J Exp Bot. 2016 Jul 19;67(17):5119–32. doi: 10.1093/jxb/erw285 (PMC5014164; doi:10.1093/jxb/erw285)
Supplement: Supplementary Data [file supp_67_17_5119__index.html]

Responses to combined abiotic and biotic stress in tomato are governed by stress intensity and resistance mechanism — Responses to combined abiotic and biotic stress in tomato are governed by stress intensity and resistance mechanism — Supplementary Data 

# Responses to combined abiotic and biotic stress in tomato are governed by stress intensity and resistance mechanism

## Supplementary Data

Data files

- supplementary\_figures\_S1\_S6\_Tables\_S1\_S2.pdf - Supplementary Data
