# Supplementary material for: Responses to combined abiotic and biotic stress in tomato are governed by stress intensity and resistance mechanism
Source: J Exp Bot. 2016 Jul 19;67(17):5119–32. doi: 10.1093/jxb/erw285 (PMC5014164; doi:10.1093/jxb/erw285)
Supplement: Supplementary Data [file supp_erw285_supplementary_figures_S1_S6_Tables_S1_S2.pdf]

Responses to combined abiotic and biotic stress in tomato are governed by stress intensity and resistance mechanism

Christos Kissoudis, Sri Sunarti, Clemens van de Wiel, Richard G.F. Visser, C. Gerard van der Linden, Yuling Bai

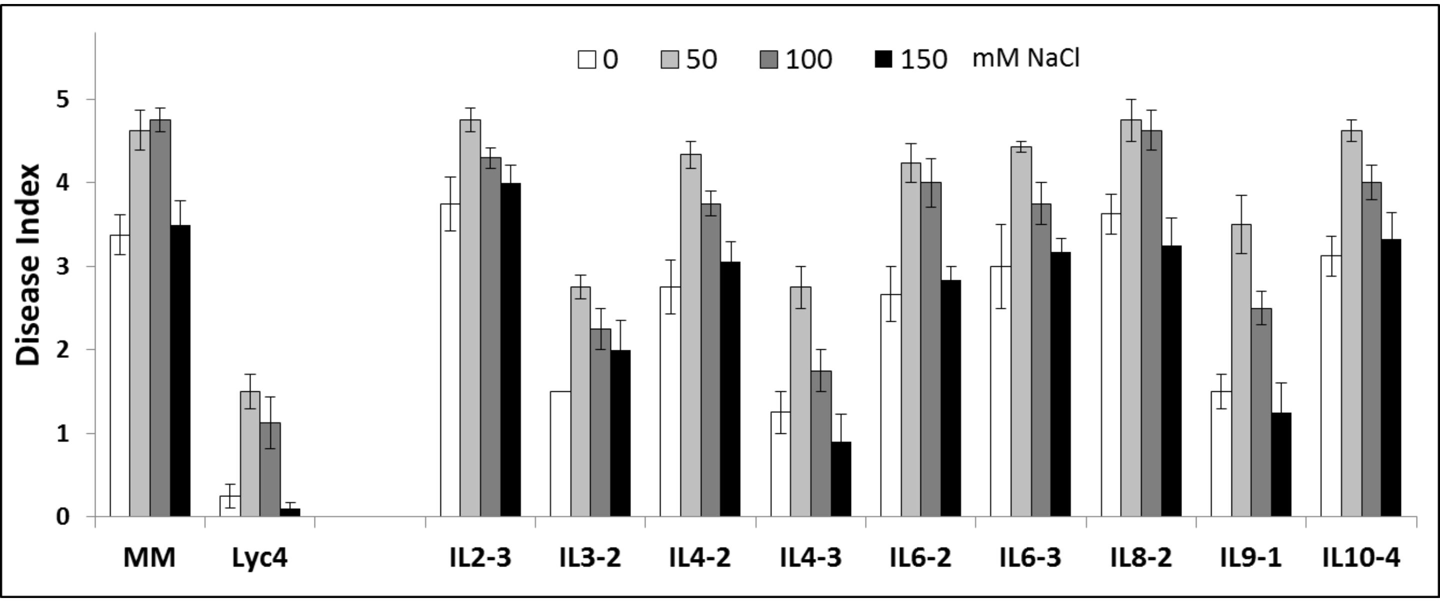

Fig. S1. Disease index of the LYC4 ILs, the recurrent parent MM and the donor parent *S. habrochaites* LYC4 under powdery mildew alone (0mM NaCl) and in combination with 50,100 and 150 mM NaCl measured at 15dpi (n=4, error bars represent standard error of mean).

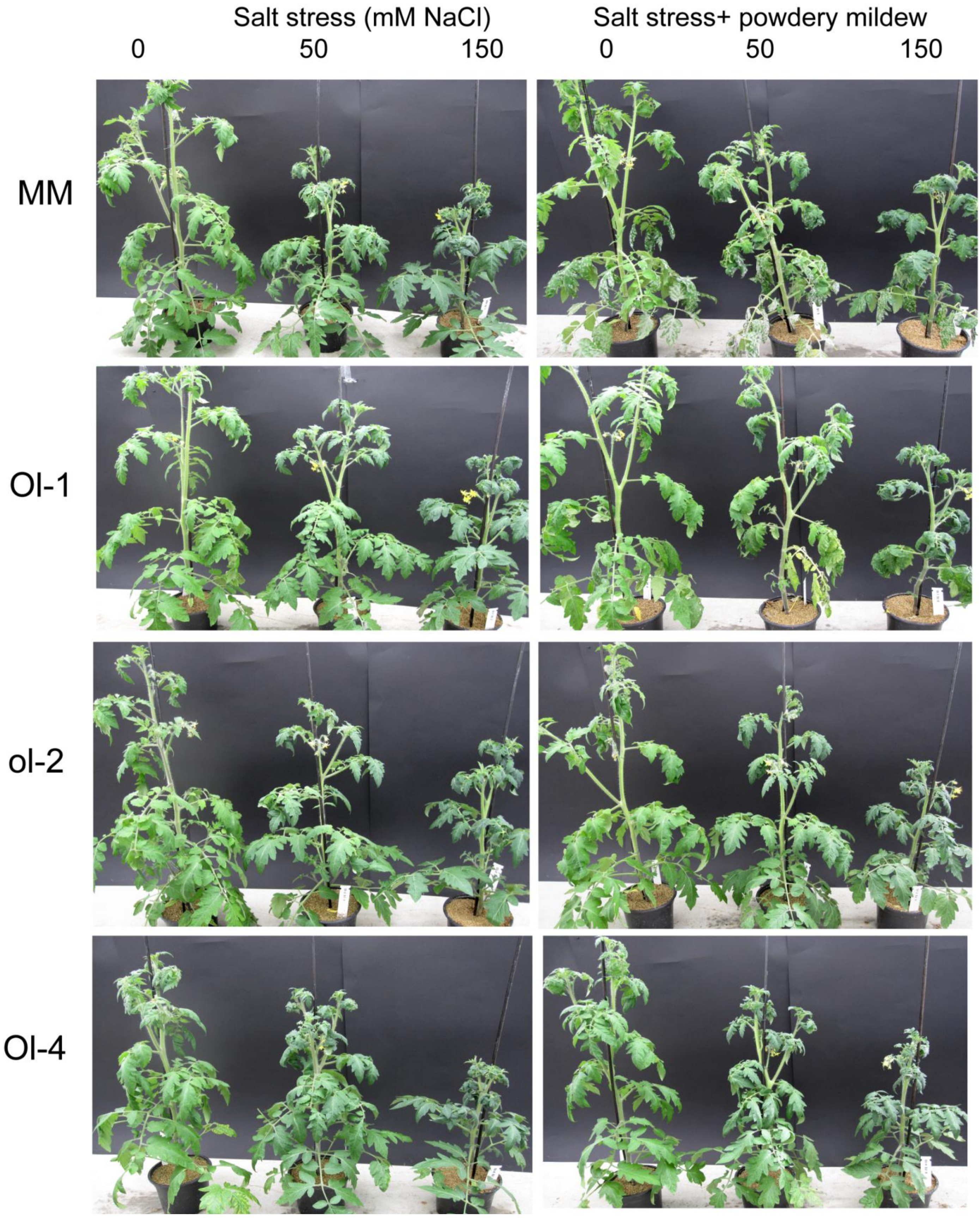

Fig. S2. Whole plant phenotypes of NILs and MM under salt stress (0, 50 150mM NaCl) alone and in combination with powdery mildew.

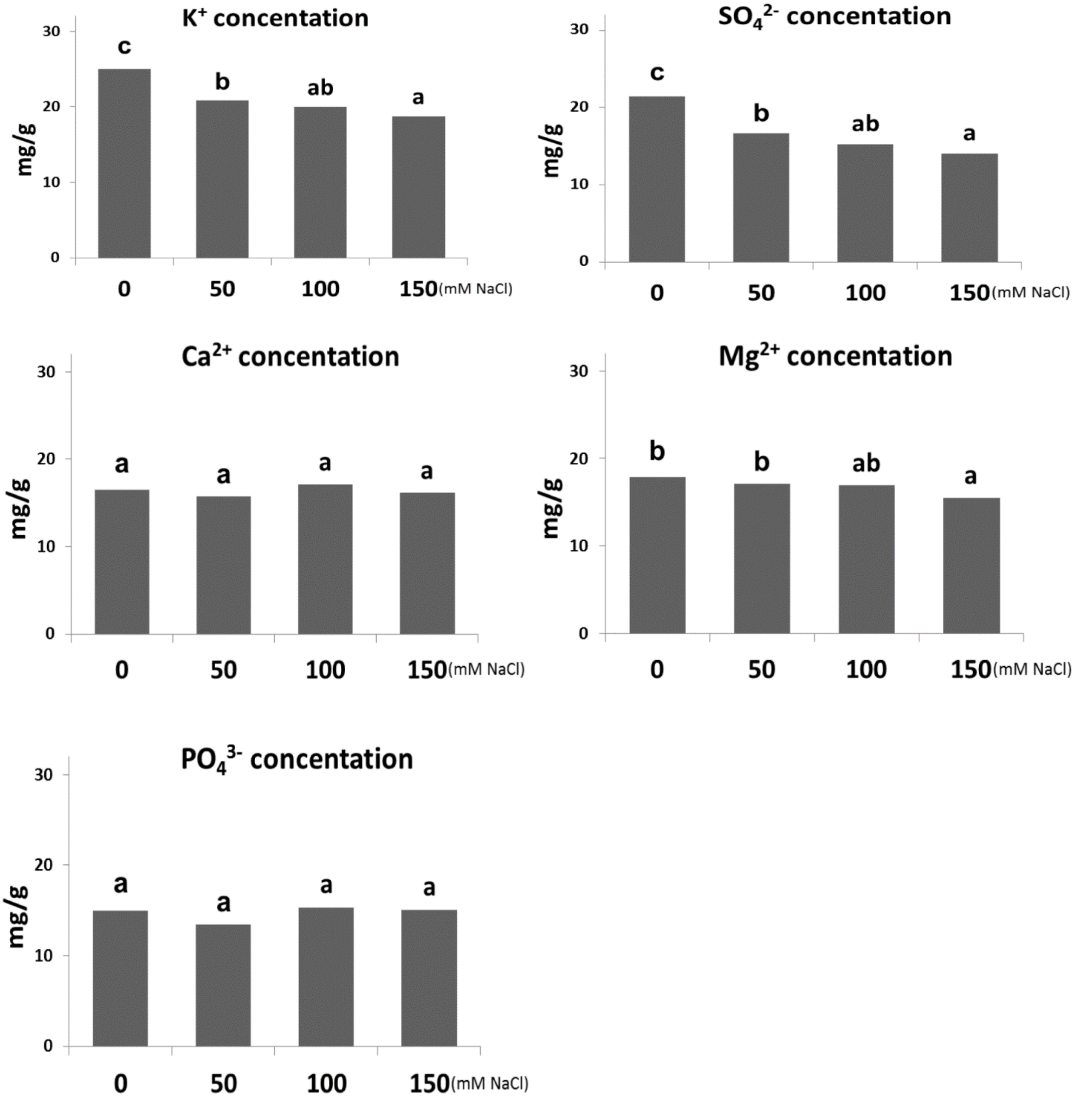

Fig. S3. Averaged K^+, SO_4^{2-}, Mg^{2+} and Ca^{2+}concentration of the LYC4 ILs and MM under powdery mildew alone (0 mM NaCl) and in combination with 50,100 and 150 mM NaCl. Statistically significant differences ($P \leq 0.05$) between treatments are designated with different letters.

	Powdery mildew					Powdery mildew+50mM NaCl					Powdery mildew+150mM NaCl				
	MM	IL3-2	IL8-2	IL9-1	Mean	MM	IL3-2	IL8-2	IL9-1	Mean	MM	IL3-2	IL8-2	IL9-1	Mean
APX	1.0	3.0	1.5	N/A	1.8 a	1.4	7.8	1.8	N/A	3.7a	4.1	2.8	5.5	N/A	4.1a
SOD	1.0	2.7	2.7	9.5	4.0 b	1.1	5.3	1.6	3.8	2.9a	1.4	1.5	4.6	1.7	2.3a
RBOHD	1.0	4.4	2.9	0.4	2.2 b	1.5	4.4	0.6	0.4	1.7ab	1.7	1.1	1.3	1.1	1.3a
RBOHF	1.0	7.7	2.6	1.0	3.1 a	0.6	5.1	4.8	1.1	2.9a	0.5	2.9	6.5	0.8	2.7a
INCED	1.0	1.2	2.4	3.7	2.1 a	1.1	3.5	1.3	4.3	2.6a	0.9	3.0	4.3	0.5	2.2a
ICS	1.0	2.3	2.0	0.8	1.5 b	1.3	1.0	1.5	1.5	1.3b	0.5	0.4	0.5	0.9	0.6a
PAL	1.0	1.4	1.8	0.8	1.2 a	0.6	6.6	1.0	1.6	2.4b	0.7	0.7	1.6	0.3	0.8a
PRI	1.0	8.7	0.7	0.6	2.7 a	9.2	14.4	4.9	1.5	7.5b	23.7	4.7	7.4	2.4	9.6b
AOS	1.0	1.0	0.9	0.9	1.0 a	2.3	3.1	0.8	2.0	2.0b	1.3	0.8	2.8	1.0	1.5ab
LOXD	1.0	3.9	1.6	0.9	1.9 a	2.4	22.4	1.1	2.0	7.0b	1.8	2.2	6.3	2.1	3.1a
ACCase	1.0	86.2	3.5	24.8	28.9 a	2.2	107.6	4.3	429.9	136.0b	2.4	66.5	10.3	32.1	27.8a
CHI	1.0	1.3	2.8	4.9	2.5 a	1.0	3.8	2.4	5.2	3.1a	1.2	2.0	2.7	1.5	1.9a
MLO	1.0	2.7	1.2	3.8	2.2 a	3.1	9.6	1.5	2.4	4.2b	2.8	2.1	1.8	1.1	1.9a

0.40 0.70 1 2 4 8 32 128 >128

Fig. S4. Gene expression heatmap of selected LYC4 ILs and the recurrent parent MM under powdery mildew stress without salt and in combination with salt stress (50,150 mM NaCl). Expression values are presented as ratios divided by the expression measured in MM under powdery mildew (=1). Statistically significant differences (P≤0.05) between treatments for each gene are designated with different letters.

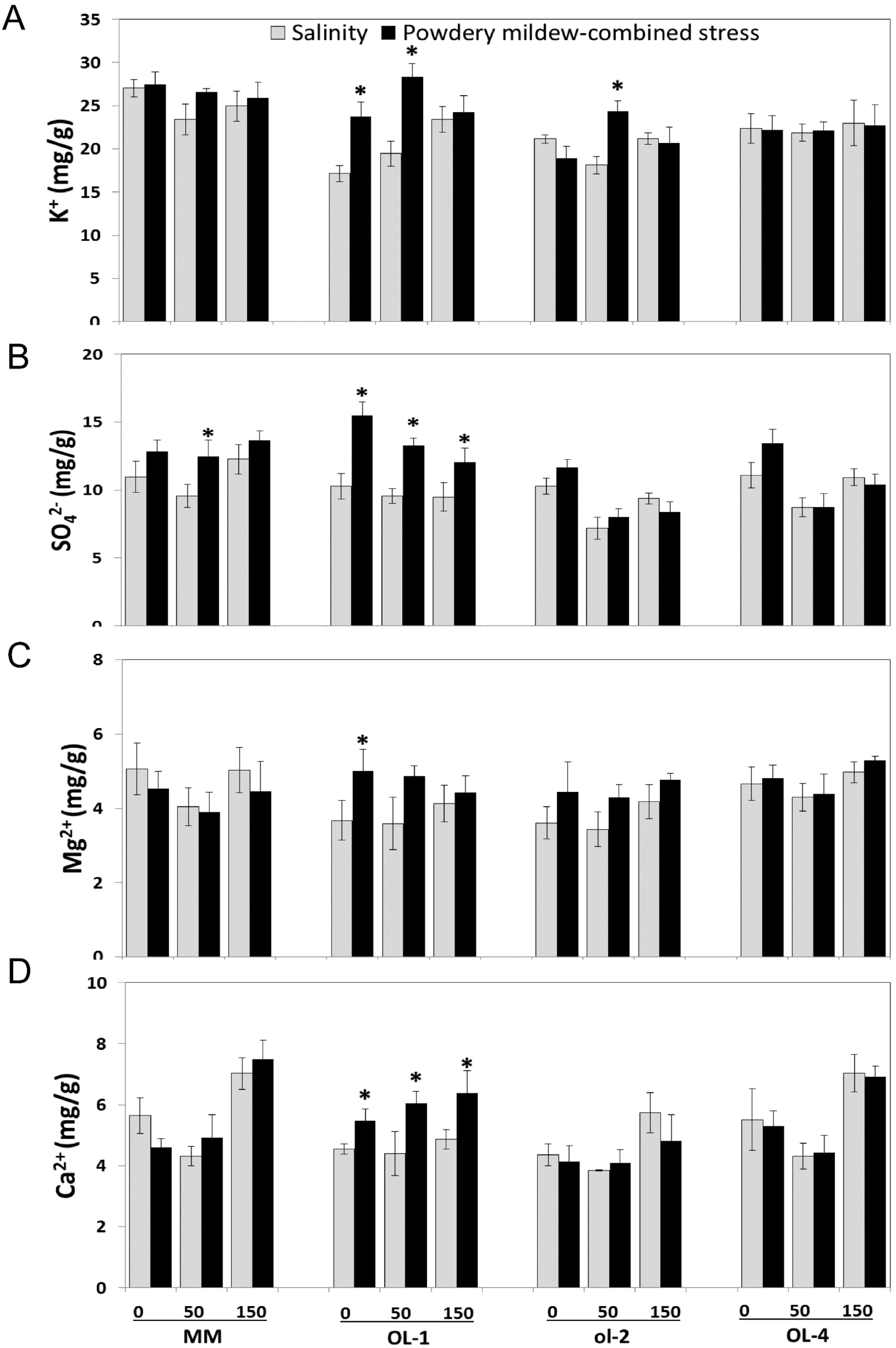

Fig. S5. K^+, SO_4^{2-}, Mg^{2+} and Ca^{2+}concentration of Ol-lines and MM. Treatment scheme as presented in Fig. 5. Asterisks denote statistically significant differences ($P \leq 0.05$) between salinity and powdery mildew-combined stress for individual genotypes (n=4, error bars represent standard error of mean).

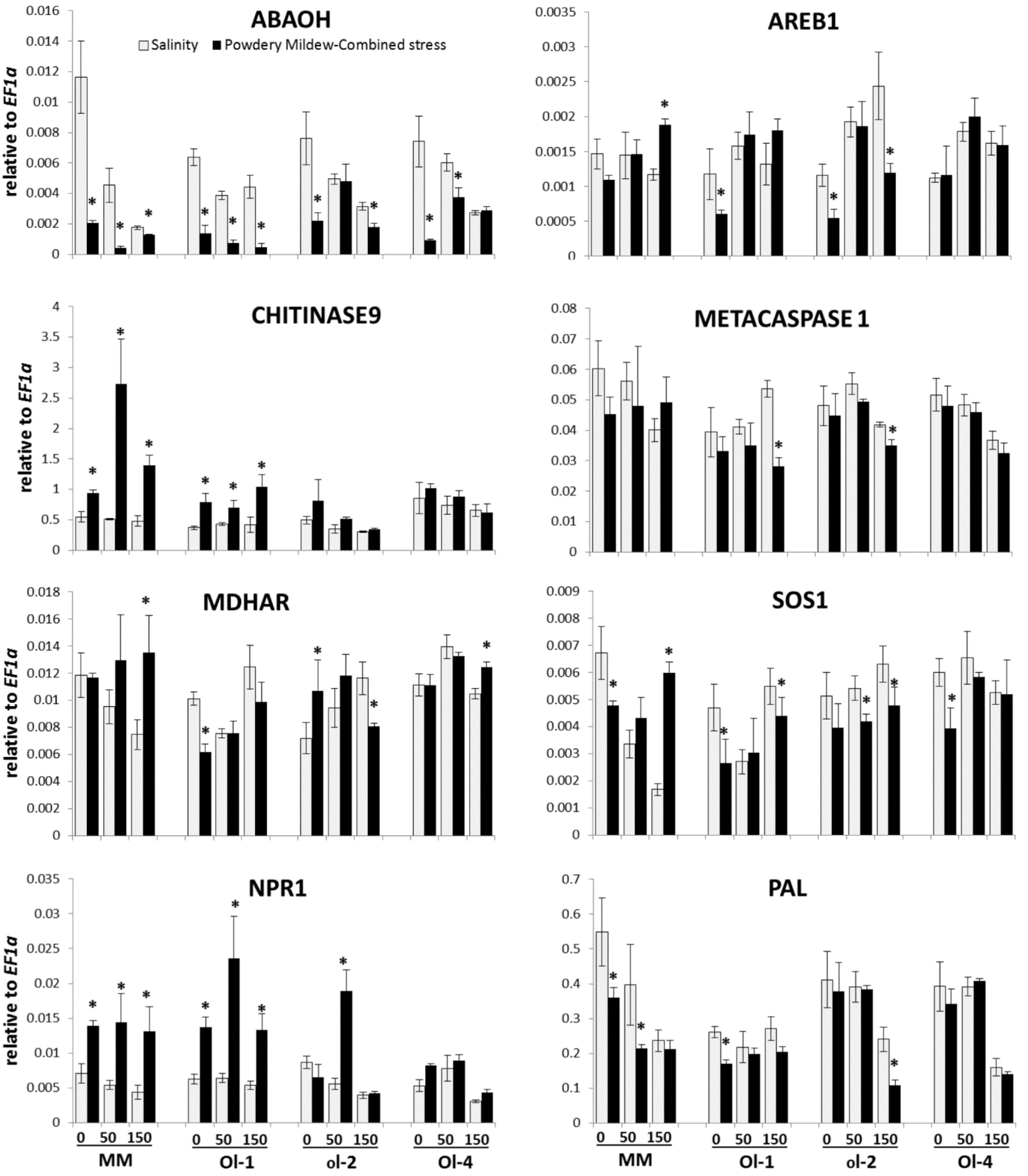

Fig. S6. Expression analysis of additional genes related to hormone and stress signalling in MM, NIL-Ol-1, -ol-2 and -Ol-4 (written as Ol-1, ol-2, Ol-4 in the figure). Treatment and labelling scheme are the same as Fig.7.

Table S1. Primers used for expression analyses with qRT-PCR.

Gene name		Sequence (5'-3')	Gene function
SlNCED1	NCED1_F	TCGAAAACCCGGATGAACAAGTGA	ABA biosynthesis
	NCED1_R	AACCAGAAACTTTTGGCCATGGTTC	
SlABAOH	ABAOH_F	TTGCTGCACAAGATACAACAGCAAG	ABA catabolism
	ABAOH_R	TGTCCATGTCAACCCATGATTTTCT	
SlAREB1	AREB1 _F	TAATTTGCCACTGAATGTGAATGGG	ABA responsive transcription factor
	AREB1_R	CCCAACTGACCACTATTTGGGATA	
SlDHN_TAS14	DHN_TAS_F	CACCATGAGGGGCAACAGCA	ABA responsive dehydrin
	DHN_TAS_R	TCACCTTCATGTTGTCCAGGCATC	
SlACCase	ACCase_F	CGCGATGAGGTTAGGTAAAAGGCA	Ethylene biosynthesis
	ACCase_R	GTCGATTCCCTTAAAAGTGGACGCA	
SlACO1	ACO1_F	TAACGGGAAGTACAAGAGTGTGC	Ethylene biosynthesis
	ACO1_R	CTTGTGTACTTTCCTCTGCCTCTT	
SlCHI9	CHI9_F	GTCATCACCGGAAGATGGCAGC	Ethylene reponsive chitinase
	CHI9_R	CCGATCCTGGACCCTGCTGT	
SlAOS	AOS_F	CCGGCGGGAAGATCACGATG	Jasmonic acid biosynthesis
	AOS_R	TCGAAAACGGCGTCGTGTGA	
SlLOXD	LOXD_F	GCAGTACCGGACGCAACACA	Jasmonic acid biosynthesis/reponse
	LOXD_R	CTGCAAACTTGGGCCGAGGA	
SlICS	ICS_F	GGCAATAGATGCACTTCAGGCCA	Salicylic acid biosynthesis
	ICS_R	CGCATGGTCCCAAGACGCTTT	
SlNPR1	NPR1_F	TGATGGCACGTCTGAATTACCC	Salicylic acid/defense resposne
	NPR1_R	AACCGTTTTCCAAGTTCCACAGTT	
SlPR1a4	SR1a4_F	GTGTCCGAGAGGCCAGACTA	Salicylic acid/defense resposne
	SR1a4_R	CATTGTTGCAACGAGCCCGA	
SlPAL	PAL_F	GCTGTCAAGAACACAGTGAGCCA	Secondary metabolism
	PAL_R	GGTAGGTGGAGCTGCAGGGA	
SlRBOHD	RBOHD_F	TCAGGTCAAGCATCAAAGCCGTT	ROS production
	RBOHD_R	TGGTGAAACCGCAGCACAGT	
SlRBOHF	RBOHF_F	GGAGTGGAGGGTGTGACTGGA	ROS production
	RBOHF_R	GGTGCGAGTACCAGAACGCA	
SlMCA1	MCA_F	CACTCTTTGACGTCTTTGGCG	Metacaspace-Cell death
	MCA_R	AACCATACCCATGAACCCGC	
SlAPX1	SlAPX1_F	CCATTTGGAACAATCAGGCACCCG	Antioxidant defence-Redox regulation
	SlAPX1_R	CGGGGCCTCCCGTAACTTCA	
SlSOD	SlSOD_F	CCTCTCACTGGTCCACAGTCCA	Antioxidant defence-Redox regulation
	SlSOD_R	AGCAGTTAACCCTGGAGGCCA	
SlMDHAR	MDHAR_F	TTGGAGCAAAACCTGCCGTC	Ascorbate regeneration
	MDHAR_R	CTGCCACATCTCCAATGGCA	
SlNHX3	SlNHX_3F	GGGTCTTGTTTTGGTTGGAAGGGC	Na+ vacuolar compartmentiation
	SlNHX3_R	CCCACTGAAACAGCACCTCGC	
SlSOS1	SlSOS1_F	GCACATCTTCACGATGCTGTCCA	Na+ cell exclusion
	SlSOS1_R	AGAGGGTTGCACCACGTAACTTCA	
SlLIN6	LIN_F	TTGGTTCAATGGCCTGTTCAAG	Cell wall invertase
	LIN_R	TTCAACGTCAGCCTGTGCAA	
SlMLO1	MLO1_F	GAAGCTGGCACCATACAGCGA	Defence reponses
	MLO1_R	CGCGTAATAAGTGAACAGGGGAGG	

Table S2. Genetic correlations of traits measured in the LYC4 ILs and the recurrent parent MM under powdery mildew individually and in combination with different levels of salt. Statistically significant correlations (P≤0.1) are highlighted.

	Ca^{2+}	1	-												
Powdery mildew	Chlorophy	2	0.1873	-											
	Cl^-	3	**-0.3979**	**-0.373**	-										Correlations
	DI10	4	-0.324	0.1552	0.1094	-									
	DW	5	-0.2178	-0.272	0.181	**-0.391**	-								
	FW	6	-0.1983	-0.267	0.3116	**-0.401**	**0.9642**	-							
	K^+	7	-0.1096	-0.266	0.355	0.0706	-0.115	-0.065	-						
	Mg^{2+}	8	**0.863**	0.271	-0.286	-0.125	-0.26	-0.262	0.2428	-					
	Na^+	9	**0.48**	0.1197	-0.026	0.2117	-0.066	-0.115	0.2929	**0.6771**	-				
	PO_4^{3-}	10	**0.8344**	0.2828	**-0.437**	-0.184	-0.249	-0.269	0.0646	**0.8518**	**0.5238**	-			
	SO_4^{2-}	11	**0.3736**	0.128	-0.229	**-0.525**	**0.4742**	**0.471**	0.1496	0.3349	0.1975	0.2974	-		
	Senescence15	12	0.2368	-0.171	-0.265	0.0301	-0.005	-0.104	-0.311	0.134	0.1646	0.177	-0.054	-	
			1	2	3	4	5	6	7	8	9	10	11	12	

	Ca^{2+}	1	-												
	Chlorophy	2	0.4038	-											
	Cl^-	3	**0.0666**	**0.0872**	-										P-values
	DI10	4	0.1413	0.4905	0.628	-									
	DW	5	0.3302	0.22	0.4203	**0.0718**	-								
	FW	6	0.3764	0.2306	0.158	**0.0645**	**<0.001**	-							
	K^+	7	0.6273	0.2316	0.1049	0.7549	0.6096	0.7737	-						
	Mg^{2+}	8	**<0.001**	0.2225	0.1977	0.5802	0.2431	0.2382	0.2762	-					
	Na^+	9	**0.0238**	0.5958	0.9103	0.3442	0.7707	0.609	0.1859	**<0.001**	-				
	PO_4^{3-}	10	**<0.001**	0.2023	**0.042**	0.4136	0.2632	0.226	0.7751	**<0.001**	**0.0123**	-			
	SO_4^{2-}	11	**0.0868**	0.5701	0.3045	**0.0121**	**0.0258**	**0.0269**	0.5064	0.1276	0.3782	0.1788	-		
	Senescence15	12	0.2887	0.4467	0.2339	0.8942	0.9841	0.6448	0.1596	0.5522	0.4641	0.4307	0.8117	-	
			1	2	3	4	5	6	7	8	9	10	11	12	

Powdery mildew	Ca^{2+}	1	-												
50mM NaCL	Chlorophy	2	-0.2361	-											Correlations
	Cl^-	3	0.0642	**0.5679**	-										
	DI10	4	**-0.4687**	0.3049	-0.09	-									
	DW	5	-0.0396	-0.093	0.1736	-0.268	-								
	FW	6	0.1138	0.1356	0.3313	-0.21	**0.8679**	-							
	K^+	7	-0.3923	**0.7527**	**0.6477**	0.2613	0.1381	0.3434	-						
	Mg^{2+}	8	**0.8877**	-0.159	0.1777	-0.298	-0.237	-0.073	-0.188	-					
	Na^+	9	0.4098	0.2707	**0.5586**	-0.192	0.3213	0.3639	0.0098	0.252	-				
	PO_4^{3-}	10	**0.6359**	-0.05	-0.219	0.0197	**-0.547**	**-0.493**	**-0.432**	**0.6461**	0.0317	-			
	SO_4^{2-}	11	**0.6864**	0.0362	0.0023	-0.38	0.1073	0.3012	-0.013	**0.6141**	0.3028	0.3827	-		
	Senescence15	12	-0.0831	-0.355	0.0628	-0.302	**0.5215**	**0.4977**	0.0425	-0.226	0.1241	**-0.504**	0.0978	-	
			1	2	3	4	5	6	7	8	9	10	11	12	

	Ca^{2+}	1	-												
	Chlorophy	2	0.3617	-											P-values
	Cl^-	3	0.8067	**0.0174**	-										
	DI10	4	**0.0577**	0.234	0.7304	-									
	DW	5	0.8801	0.7221	0.5052	0.2989	-								
	FW	6	0.6636	0.6038	0.1939	0.4176	**<0.001**	-							
	K^+	7	0.1194	**<0.001**	**0.0049**	0.3111	0.5972	0.1772	-						
	Mg^{2+}	8	**<0.001**	0.5417	0.4951	0.2456	0.3594	0.7817	0.4701	-					
	Na^+	9	0.1023	0.2934	**0.0198**	0.4602	0.2086	0.151	0.9701	0.3293	-				
	PO_4^{3-}	10	**0.0061**	0.8502	0.3987	0.9403	**0.0231**	**0.0444**	**0.0837**	**0.0051**	0.9039	-			
	SO_4^{2-}	11	**0.0023**	0.8903	0.9929	0.1324	0.6819	0.24	0.9596	**0.0087**	0.2375	0.1295	-		
	Senescence15	12	0.7511	0.1616	0.8106	0.2394	**0.0318**	0.0421	0.8715	0.3834	0.6352	**0.039**	0.7088	-	
			1	2	3	4	5	6	7	8	9	10	11	12	

Table S2 (cont.)

Powdery mildew 100mM NaCL

Correlations

Ca^{2+}	1	-											
Chlorophy	2	0.0824	-										
Cl^-	3	0.2795	0.1283	-									
DI10	4	-0.0782	-0.063	0.0509	-								
DW	5	-0.0793	-0.171	**-0.351**	-0.106	-							
FW	6	-0.0448	-0.071	-0.172	-0.22	**0.7563**	-						
K^+	7	**-0.3877**	0.2289	0.0902	-0.205	0.0398	0.0169	-					
Mg^{2+}	8	**0.76**	0.1944	**0.4996**	-0.034	**-0.43**	**-0.422**	-0.053	-				
Na^+	9	0.044	-0.142	**0.7575**	0.2745	-0.181	0.0438	-0.15	0.0927	-			
PO_4^{3-}	10	**0.6903**	-0.057	**0.4472**	0.1414	**-0.496**	-0.378	-0.21	**0.8009**	0.2062	-		
SO_4^{2-}	11	**0.3848**	0.0342	-0.002	-0.03	**0.3583**	0.21	-0.045	0.1975	0.026	0.115	-	
Senescence15	12	**-0.3555**	-0.166	-0.176	-0.239	**0.3895**	0.2101	0.271	**-0.364**	-0.1054	**-0.398**	0.0883	-
		1	2	3	4	5	6	7	8	9	10	11	12

P-values

Ca^{2+}	1	-											
Chlorophy	2	0.6433	-										
Cl^-	3	0.1094	0.4697	-									
DI10	4	0.6603	0.7229	0.7751	-								
DW	5	0.6557	0.3331	0.0416	0.5497	-							
FW	6	0.8012	0.6913	0.3316	0.2123	**<0.001**	-						
K^+	7	**0.0235**	0.193	0.6119	0.244	0.8232	0.9242	-					
Mg^{2+}	8	**<0.001**	0.2705	**0.0026**	0.8496	**0.0112**	**0.0129**	0.7648	-				
Na^+	9	0.8049	0.4235	**<0.001**	0.1161	0.3065	0.8057	0.3958	0.602	-			
PO_4^{3-}	10	**<0.001**	0.7479	**0.008**	0.4249	**0.0029**	**0.0276**	0.2342	**<0.001**	0.2419	-		
SO_4^{2-}	11	**0.0246**	0.8477	0.9929	0.8645	**0.0375**	0.2333	0.7995	0.2628	0.8837	0.5173	-	
Senescence15	12	**0.0391**	0.3482	0.3186	0.1729	**0.0228**	0.2331	0.1211	**0.0344**	0.5529	**0.0198**	0.6196	-
		1	2	3	4	5	6	7	8	9	10	11	12

Powdery mildew 150mM NaCL

Correlations

Ca^{2+}	1	-											
Chlorophy	2	-0.0804	-										
Cl^-	3	**0.5101**	-0.171	-									
DI10	4	-0.1144	0.1558	0.2119	-								
DW	5	**-0.5274**	0.0779	**-0.494**	-0.171	-							
FW	6	**-0.4371**	0.0901	**-0.457**	-0.065	**0.8182**	-						
K^+	7	-0.1165	0.1993	**0.3347**	0.2075	0.1709	0.0646	-					
Mg^{2+}	8	**0.7924**	-0.057	**0.4127**	-0.109	**-0.412**	**-0.341**	-0.013	-				
Na^+	9	**0.4062**	**-0.357**	**0.8087**	0.147	**-0.33**	**-0.339**	0.1444	0.1411	-			
PO_4^{3-}	10	**0.706**	-0.245	**0.2911**	0.0652	**-0.588**	**-0.439**	-0.183	**0.6327**	0.2637	-		
SO_4^{2-}	11	0.2482	0.176	-0.071	-0.315	0.2293	0.2289	0.1723	0.2018	0.0611	-0.05	-	
Senescence15	12	-0.1417	**-0.388**	**-0.38**	**-0.467**	**0.3831**	**0.4348**	-0.102	-0.238	-0.1239	-0.14	**0.344**	-
		1	2	3	4	5	6	7	8	9	10	11	12

P-values

Ca^{2+}	1	-											
Chlorophy	2	0.6461	-										
Cl^-	3	**0.0017**	0.3261	-									
DI10	4	0.5129	0.3716	0.2217	-								
DW	5	**0.0011**	0.6566	**0.0026**	0.3265	-							
FW	6	**0.0087**	0.6067	**0.0058**	0.7125	**<0.001**	-						
K^+	7	0.5051	0.2511	**0.0494**	0.2316	0.3264	0.7123	-					
Mg^{2+}	8	**<0.001**	0.7472	**0.0137**	0.5343	**0.014**	**0.045**	0.942	-				
Na^+	9	**0.0155**	**0.0351**	**<0.001**	0.3993	**0.0532**	**0.0462**	0.4079	0.4188	-			
PO_4^{3-}	10	**<0.001**	0.1554	**0.0897**	0.7099	**<0.001**	**0.0083**	0.2923	**<0.001**	0.1258	-		
SO_4^{2-}	11	0.1506	0.312	0.6861	0.0656	0.1851	0.186	0.3224	0.245	0.7272	0.7749	-	
Senescence15	12	0.4167	**0.0213**	**0.0243**	**0.0047**	**0.0231**	**0.0091**	0.5614	0.1683	0.4782	0.422	**0.043**	-
		1	2	3	4	5	6	7	8	9	10	11	12
